# Efficacy of computed tomography-assisted limited decompression in the surgical management of thoracolumbar fractures with neurological deficit

**DOI:** 10.1186/s13018-021-02388-4

**Published:** 2021-04-14

**Authors:** Landa Shi, Dean Chou, Yuqiang Wang, Mirwais Alizada, Yilin Liu

**Affiliations:** 1 Department of the Orthopaedic Surgery, The First Affiliated Hospital of Zhengzhou University, No.1 Jianshe East Road, Zhengzhou, China; 2 Department of the Neurosurgery, University of California San Francisco, San Francisco, CA USA

**Keywords:** CT-assisted, Thoracolumbar fractures, Precision medicine, ERAS, Limited decompression

## Abstract

**Objective:**

To investigate the effect of CT-assisted limited decompression in managing single segment A3 lumbar burst fracture.

**Method:**

A retrospective study (January 2015–June, 2019). One hundred six cases with single-level Magerl type A3 lumbar burst fractures treated with short-segment posterior internal fixation and limited decompression. Patients were divided into two groups: CT-assisted group and non-CT-assisted group. Perioperative factors, clinical outcomes, post-operative complications, imaging parameters, and health-related quality of life (HRQoL) were evaluated.

**Results:**

Kyphosis, loss of anterior and posterior vertebral body heights, operative time, and post-operative complications were not significantly different between the two groups. The visual analog score (VAS) and spinal canal encroachment in the CT-assisted group were lower compared with the non-CT-assisted group *(p* < 0.05). The Japanese Orthopaedic Association (JOA) score, the simplified HRQoL scale, and the American Spinal Injury Association (ASIA) Spinal Cord Injury Grade in the CT-assisted group were significantly higher compared with the non-CT-assisted group *(p* < 0.05).

**Conclusion:**

CT-assisted limited decompression in the treatment of single-segment A3 lumbar burst fracture can achieve better fracture reduction and surgical results and improve the long-term recovery of the patients’ neurological function and quality of life.

## Introduction

Many spinal column fractures occur in the thoracolumbar region, with three column injuries or burst fractures. It is generally accepted that AO-Magerl Type B and C [1] thoracolumbar fractures should be treated surgically. Anterior or anterior and posterior combined approaches can achieve adequate decompression and reconstruction. However, with the improvement of medical standards and the accumulation of experience, the posterior approach has been fully developed. The posterior approach reduces the injured vertebra to restore the normal height and reinforce the vertebral body’s strength with bone grafting. For type A3 thoracolumbar burst fractures with neurologic deficits, the traditional posterior decompression technique can alleviate dural sac compression, restore height, and correct kyphosis. However, the posterior ligamentous complex and zygapophysial joints often need to be excised [2, 3].

Consequently, the posterior column is disrupted, further destabilizing the spinal column and increasing the risk of post-operative kyphosis, implant failure, or neurologic symptoms [4]. In recent years, spine surgeons have gradually chosen pedicle screw reduction combined with interlaminar fenestration decompression. Bilateral lamina fenestration decompression is more thorough in nerve root decompression, which improves the surgical effect, while unilateral lamina decompression can better maintain the bony structure’s integrity and effectively improve the posterior column’s ability to bear the torsional force. It is inevitable to know how to balance meticulous nerve decompression and minor spinal column injury. If we can timely evaluate the degree of spinal canal’s posterior wall reduction during the operation and determine the residual bone mass position, bilateral lamina fenestration can be avoided under the premise of thorough decompression. C-arm fluoroscopy and mobile C-arm CT cannot reconstruct coronal, sagittal, or arbitrarily angled planes to fully display the fracture line and the fracture’s displacement, increasing the cross-sectional false-negative phenomenon [5]. Some scholars found that post-operative CT scan of some patients with unilateral lamina decompression still showed residual bone masses in the spinal canal, and the spinal canal diameter did not return to the normal or close to the normal level [5]. A hybrid operating room (HOR) with multi-slice spiral computed tomography (CT) can be used to delineate the surgical correction further and overcome the obstacles mentioned above. We evaluated our experience using HOR to treat the CT-assisted group and the non-CT assisted group patients of type A3 burst fractures with neurologic symptoms. Our goal was to assess the efficacy of individualizing a precise surgical procedure for each patient [6, 7].

## Methods

### Patients

Patients who underwent limited decompression and internal fixation of type A3 burst fractures with pedicle screw fixation by the senior surgeon in our spine surgery center from January 2015 to June 2019 were retrospectively reviewed. All patients had a minimum of a 12-month follow-up. The inclusion criteria were (1) type A3 single-segment lumbar burst fracture, (2) neurologic deficit, (3) absence of major medical comorbidities, (4) treatment within 7 days of the injury, and (5) an intact posterior ligamentous complex. The exclusion criteria were (1) severe osteoporosis, (2) pathological fracture caused by infection and tumor, (3) injury of the posterior ligamentous complex, and (4) inability to comply with treatment recommendations. The Ethics Committee of The First Affiliated Hospital of Zhengzhou University approved this study. Written informed consents were obtained from all the participants.

### Surgical technique

**Fig. 1:**
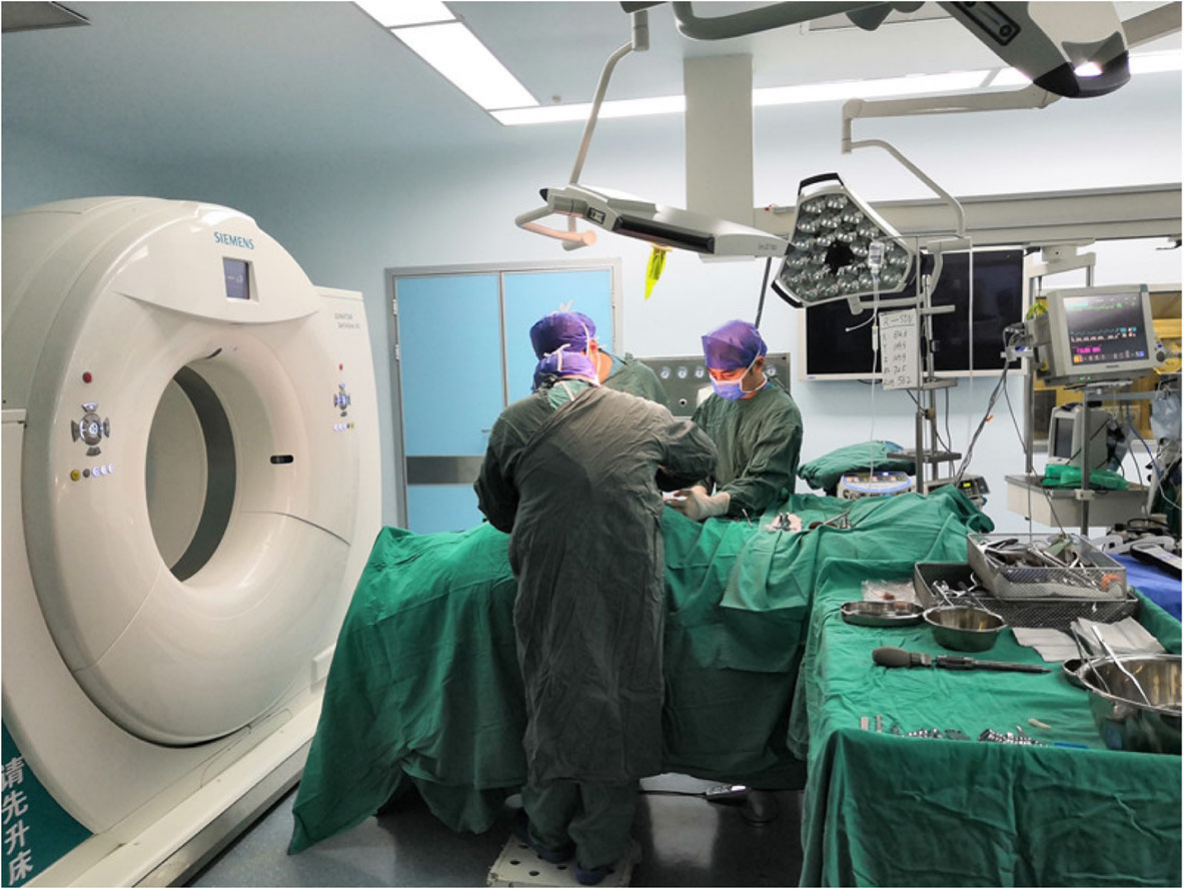
Photos of surgical procedures. After general anesthesia was induced, the patient was placed prone. A posterior midline approach was performed. One caudal adjacent and one cephalad adjacent spines to the fractured vertebra were exposed. The spinous processes and supraspinous ligaments were preserved. Pedicle screws with extension tabs were placed in adjacent spines above and below the fractured vertebra in preparation for ligamentotaxis using the posterior longitudinal ligament (PLL). Two straight rods were placed with set screws, one on each side. The vertebrae were distracted, and the set screws were tightened. The straight rods were subsequently replaced with lordotic-contoured rods one at a time to match physiologic lordosis. The thoracolumbar segment is the transitional area of the spine from thoracic kyphosis to lumbar lordosis. When dealing with L1 fracture, there is no need to contour the rod; the straight rod can solve the problem. In the CT-assisted group, an intraoperative CT scan was performed to assess the extent of spinal canal clearance by ligamentotaxis. A limited decompression of unilateral lamina was performed. The fractured vertebral body’s posterior wall was tamped ventrally (Intraoperative CT-scanning was performed to track and check whether the spinal canal’s posterior wall is reduced close to normal determine the residual bone mass position. It plays a decisive guiding role in the operation and avoids bilateral lamina fenestration under the premise of meticulous decompression). In the non-CT assisted group, bilateral limited lamina decompression was performed to obtain more thorough nerve decompression, which is beneficial to improve the surgical effect. The wound was irrigated, a drain was placed on each side, and the wound was closed in layers. The lateral portions of the facet joints directly adjacent to the transverse processes and the facet joints themselves of both groups’ fractured vertebrae were decorticated with a pneumatic burr until bleeding cancellous bone was visible. A composite artificial bone graft was placed along the decorticated lateral margin of the facet joints as well as within the facet joints

After general anesthesia was induced, the patient was placed prone. A posterior midline approach was performed. One caudal adjacent and one cephalad adjacent spines to the fractured vertebra were exposed. The spinous processes and supraspinous ligaments were preserved. Pedicle screws with extension tabs were placed in adjacent spines above and below the fractured vertebra in preparation for ligamentotaxis using the posterior longitudinal ligament (PLL). Two straight rods were placed with set screws, one on each side. The vertebrae were distracted, and the set screws were tightened. The straight rods were subsequently replaced with lordotic-contoured rods one at a time to match physiologic lordosis. The thoracolumbar segment is the transitional area of the spine from thoracic kyphosis to lumbar lordosis. When dealing with L1 fracture, there is no need to contour the rod; the straight rod can solve the problem. In the CT-assisted group, an intraoperative CT scan was performed (Fig. 1) to assess the extent of spinal canal clearance by ligamentotaxis. A limited decompression of unilateral lamina was performed. The fractured vertebral body’s posterior wall was tamped ventrally (intraoperative CT-scanning was performed to track and check whether the spinal canal’s posterior wall is reduced close to normal determine the residual bone mass position. It plays a decisive guiding role in the operation and avoids bilateral lamina fenestration under the premise of meticulous decompression). In the non-CT assisted group, bilateral limited lamina decompression was performed to obtain more thorough nerve decompression, which is beneficial to improve the surgical effect. The wound was irrigated, a drain was placed on each side, and the wound was closed in layers. The lateral portions of the facet joints directly adjacent to the transverse processes and the facet joints themselves of both groups’ fractured vertebrae were decorticated with a pneumatic burr until bleeding cancellous bone was visible. A composite artificial bone graft was placed along the decorticated lateral margin of the facet joints as well as within the facet joints. The senior author performed all surgeries.

### Data collection and outcome measures

Demographic variables, perioperative factors, complications, clinical outcomes, and radiographic data were collected. Pain level was assessed by the visual analog score (VAS) [8]. The Japanese Orthopedic Association (JOA) score [9] was used to assess the lower extremities’ neurological function. The short form 12 questionnaire (SF-12) [10] was used to assess health-related quality of life (HRQoL), and the American Spinal Injury Association (ASIA) scale was used to assess neurological recovery. All patients underwent anteroposterior (AP) view and lateral view radiographs preoperatively and at 3 months, 6 months, and 12 months after the surgery. Pre-operative CT and MRI were performed to evaluate the spine, and CT was performed at 3 months, 6 months, and 12 months post-operatively. The thoracolumbar sagittal Cobb angle, the percentage of anterior height loss, the percentage of posterior height loss, the percentage of spinal canal encroachment, and the mean sagittal diameters of the adjacent upper and lower vertebral bodies were compared preoperatively and at 3 months, 6 months, and 12 months post-operatively. All clinical and radiographic data were collected preoperatively and during the post-operative follow-up. All the imaging parameters were measured by the same researcher twice, and the average was calculated. All patients have been followed up for at least 12 months.

### Statistical analyses

The SPSS Version 25.0 K for Windows (SPSS, Chicago, IL) was used for the analysis. Intergroup comparisons were made using the rank-sum test (Mann-Whitney test), repeated measures analysis of variance (RM ANOVA), chi-squared test, and Fisher’s exact test. Pre-operative and post-operative clinical and radiologic outcomes were compared using the Wilcoxon Singed-rank test or paired *t* test. Values of *p< 0.05* were considered statistically significant.

## Results

### Clinical data analysis

A total of 106 patients (mean age 34.34±6.54 years; range, 18–48 years) were included in this study. The follow-up period ranged from 12 to 48 months (average 22.0 months), with all patients having a minimum of 1 year follow-up. There was no significant difference in age, regions of surgery, and gender between the CT-assisted and the non-CT assisted groups (*p* > 0.05, Table 1). There were no significant differences in comorbidities, smoking history, follow-up period, and body mass index (BMI) between the two groups (*p* > 0.05).

**Table 1:** Patient demographic data

	Non-CT-assisted group (*N*=50)	CT-assisted group (*N*=56)	*p* value
Age (mean ± SD)	33.96±6.86	34.68±6.28	0.574^*^
Sex (*n*)			
Male	30	36	0.650^φ^
Female	20	20	
Operative level			1.000^*^
L1	23	26	
L2	18	20	
L3	9	10	
Diabetes	6	8	0.729^φ^
Hypertension	4	6	0.885^φ^
Active smokers	16	18	0.987^φ^
BMI	23.66±1.64	23.70±1.61	0.900^*^

*BMI* body mass index

^*^Using the rank sum test (Mann-Whitney test)

^φ^Using chi-squared test

### Surgical outcomes

In the CT-assisted group, average intraoperative blood loss was 52.64±6.35 ml, mean post-operative drainage volume was 27.70±9.11 ml, and mean hospital stay was 9.41±1.22 days. In the non-CT-assisted group, the mean intraoperative blood loss was 52.28±6.50 ml, average post-operative drainage volume was 27.88±9.58 ml, and the mean hospital stay was 9.38±1.19 days. There was no significant difference in perioperative parameters between the two groups (*p* > 0.05) (Table 2).

**Table 2:** Patient perioperative parameters

	Non-CT-assisted group (*N*=50)	CT-assisted group (*N*=56)	*p* value
Operative time (min)	63.58±7.87	63.98±7.98	0.796*
Blood loss (ml)	52.28±6.50	52.64±6.35	0.774*
Hospital stay (days)	9.38±1.19	9.41±1.22	0.899*
Postoperative drainage (ml)	27.88±9.58	27.70±9.11	0.921*
Postoperative fever (*n*)	2	3	1.000φ
Wound infection (*n*)	2	2	1.000φ
Wound healing rate (%)	100%	100%	-

^*^Using the rank sum test (Mann-Whitney test)

^φ^Using chi-squared test

### Clinical outcomes

**Fig. 2:**
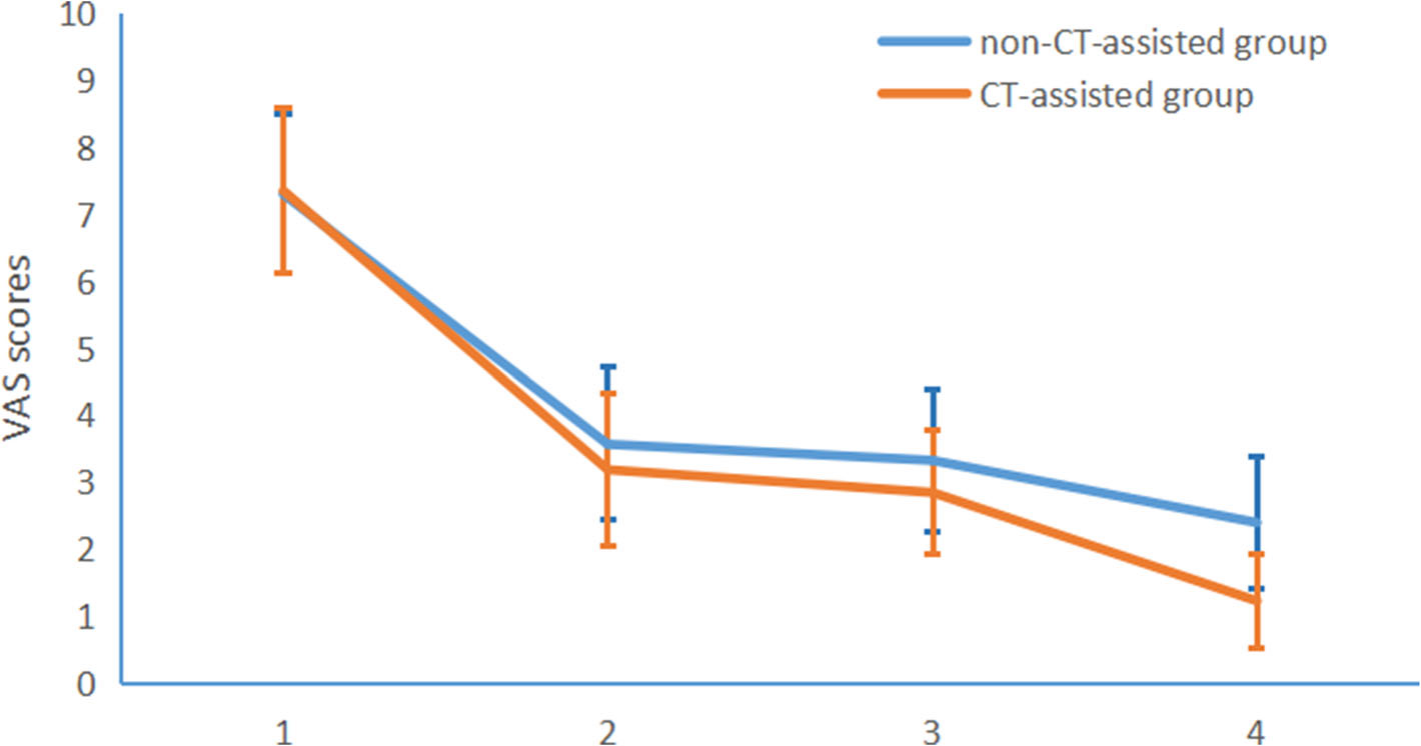
The VAS score of the CT-assisted group at 12 months was significantly lower than that of the non-CT-assisted group

**Fig. 3:**
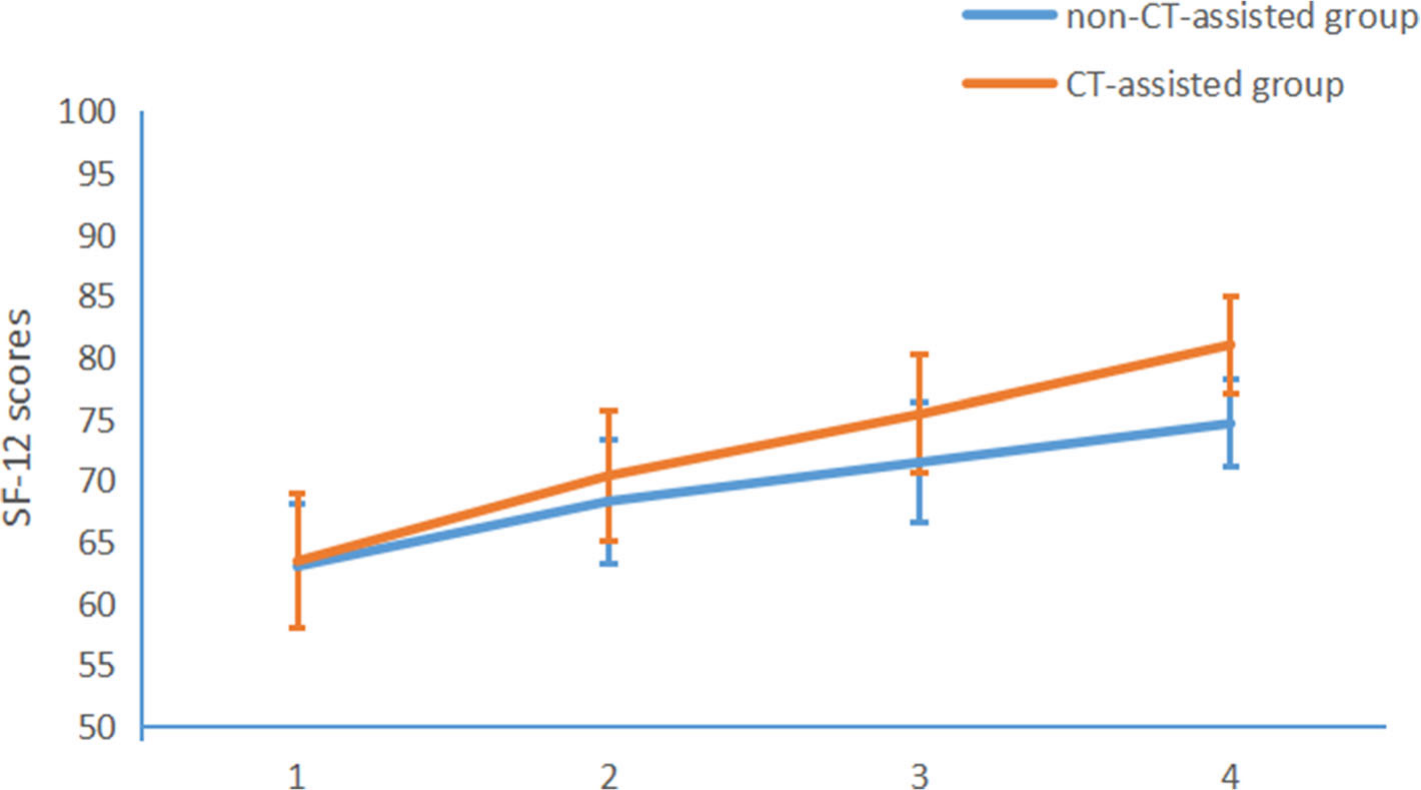
The SF-12 score of the CT-assisted group at 12-month follow-up was significantly higher than that of the non-CT-assisted group

The JOA and SF-12 scores were significantly higher in both groups at 12 months after surgery compared to the pre-operative period (*p* < 0.05). VAS scores in both groups were significantly lower post-operatively than pre-operative time (*p* < 0.05). There were no significant differences in JOA, VAS, and SF-12 scores between the two groups preoperatively (*p* > 0.05), but the VAS scores of the CT-assisted group at 12 months was 1.21±0.71, which was significantly lower than that of the non-CT-assisted group (2.40±0.99, *p* < 0.05). The SF-12 scores of the CT-assisted group at 12 months follow-up was 81.21±3.55, which was significantly higher than that of the non-CT assisted group (*p* < 0.05). The CT-assisted group patients had significantly higher HRQoL and lower VAS scores (Figs. 2 and 3). The JOA scores and long-term neurological recovery (ASIA grades) were higher in the CT-assisted group than in the non-CT-assisted group (*p* < 0.05, Table 3).

**Table 3:** Clinical outcomes between the two groups

	Non-CT-assisted group (*N*=50)	CT-assisted group (*N*=56)	*p* value
JOA scores			
Preoperative	16.10±1.56	15.89±0.97	0.049 #
Postoperative 3 months	21.46±1.11	22.34±1.39	
Postoperative 6 months	22.76±1.08	23.23±1.32	
Postoperative 12 months	23.28±0.93*	24.64±1.02*	
VAS scores			
Preoperative	7.30±1.22	7.29±1.23	0.008#
Postoperative 3 months	3.56±1.16	3.16±1.16	
Postoperative6 months	3.32±1.06	2.80±0.92	
Postoperative 12 months	2.40±0.99*	1.21±0.71**	
SF-12 scores			
Preoperative	63.36±4.89	63.34±5.32	0.000#
Postoperative 3 months	68.36±4.89	70.30±5.21	
Postoperative 6 months	71.46±4.77	75.18±4.74	
Postoperative 12 months	74.16±2.99φ	81.21±3.55φφ	

^*,*,φ^Using the paired *t* test; ^#^Using the repeated measures analysis of variance (RMANOVA). The corresponding *p* values of the JOA score, VAS score, and SF-12 score at the final follow-up of two groups compared with preoperation respectively were *p<0.05, **p<0.05, ^*^p<0.05, ^**^p<0.05, ^φ^*p*<0.05, ^φφ^*p*<0.05. *JOA* Japanese Orthopaedic Association, *VAS* Visual Analogue Scale, *SF-12* Short Form-12 Questionnaire

### Radiographic analysis

**Fig. 4:**
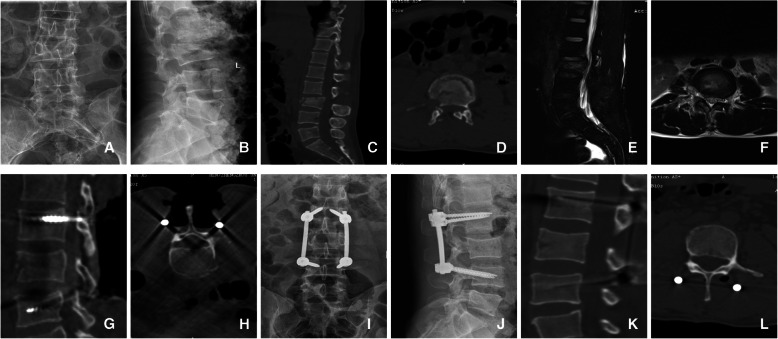
A 23-year-old male presented with a burst fracture of the L 3 vertebral body. Severe back pain with limited mobility and left lower limb muscle strength was grade II. **a**–**f** The preoperative imaging data, **g**, **h** the intraoperative CT scanning data, and **i**–**l** the 12-month postoperative imaging data. Preoperative CT scan showed the fracture fragment burst into the spinal canal (**c**, **d**), and MRI showed the dural sac was compressed (**e**, **f**).With the assistance of the intraoperative CT, a limited decompression of unilateral lamina was performed, the diameter of spinal canal returned to normal and the pressure of dural sac disappeared (**g**, **h**), the result was satisfactory (**i**-**l**) 12 months after the operation, and the muscle strength of left lower limb was grade V

**Fig. 5:**
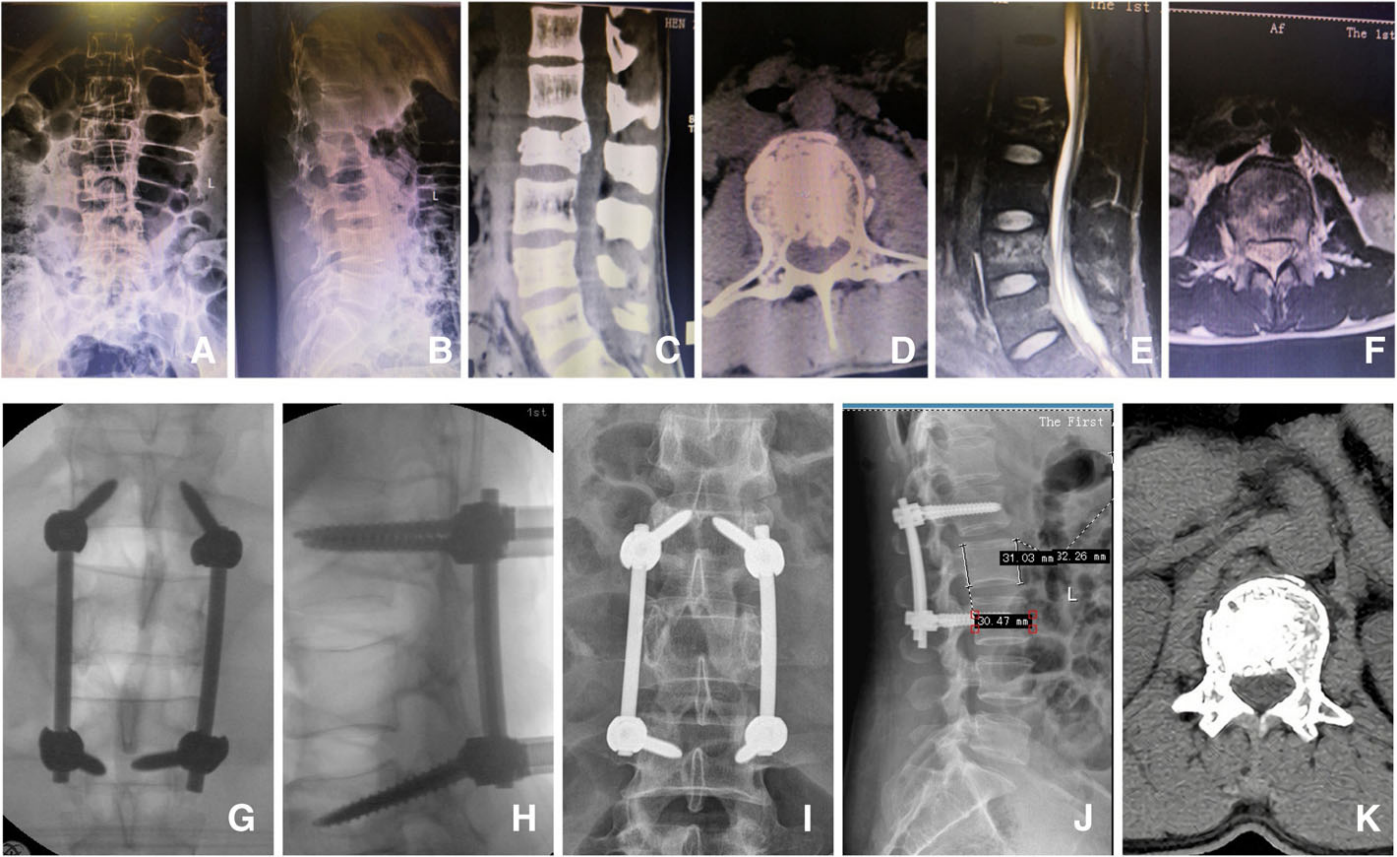
A 42-year-old male presented with a burst fracture of the L2 vertebra. Low back pain from a high fall with limited mobility and both lower limbs’ muscle strength was grade-III. **a**–**f** The preoperative images. **g**–**h** C-arm fluoroscopy after bilateral limited decompression. **i**–**k** The postoperative imaging data at a 12-month follow-up visit. Preoperative imaging data show the fractures compressed the dural sac (**c**, **d**, **e**, **f**), and bilateral limited decompression was performed after multiaxial pedicle screw fixation. At the 12-month postoperative visit, the fracture healing is satisfactory (**i**–**k**), and the muscle strength of both lower limbs was grade V

The sagittal Cobb angles, anterior and posterior vertebral body heights, and the spinal canal encroachment significantly improved 12 months after the surgery in both groups (*p*<0.05, Tables 4 and 5). There was no significant difference in the Cobb angles and vertebral body heights at 12 months post-operative between the two groups (*p* > 0.05). The spinal canal encroachment was lower in the CT-assisted group 1 year post-operative (*p*<0.05). Images of typical cases are shown in Figs. 4 and 5. In Fig. 4, a 23-year-old male presented with a burst fracture of the L3 vertebral body. The patient complained of severe back pain with limited mobility, and the left lower limb muscle strength was grade II. The pre-operative CT scan showed the fracture fragment burst into the spinal canal, and the MRI showed a compressed dural sac. With the assistance of the intraoperative CT, a limited decompression of unilateral lamina was performed, the diameter of the spinal canal returned to normal, and the pressure on the dural sac disappeared; 12 months after the operation, the muscle strength of the left lower limb returned to grade V. In Fig. 5, a 42-year-old male presented with a burst fracture of the L2 vertebra. Low back pain from a high fall with limited mobility and both lower limbs muscle strength was grade III. Pre-operative imaging data showed the fractured bone fragments compressed the dural sac. Limited bilateral decompression was performed after multiaxial pedicle screw fixation. At the 12-month post-operative visit, the CT scan showed that there was still a slight invasion in the spinal canal, but fortunately, the muscle strength of the lower limbs was V.

**Table 4:** Comparison of the grade of ASIA spinal nerve injury between groups

ASIA grade	Non-CT-assisted group (*N*=50)	CT-assisted group (*N*=56)	*p* value
Preoperative			
C	33	37	0.994*
D	17	19	
E	0	0	
Final follow-up			
C	0	0	0.030*
D	8	2	
E	42*	54**	

^*^Using the rank sum test (Mann-Whitney test). ASIA grade at the final follow-up of two groups compared with preoperation respectively were ^*^p<0.05, ^**^p<0.05

**Table 5:** Comparisons of radiographic results between groups

Variable	Non-CT-assisted group (*N*=50)	CT-assisted group (*N*=56)	*p* value
Cobb angle(°)			
Preoperative	29.56±4.10	29.46±4.01	0.841ψ
Postoperative 3 months	3.53±1.40	3.45±1.35	
Postoperative 6 months	5.45±1.22	5.36±1.22	
Postoperative 12 months	7.30±1.37*	7.30±1.38**	
Percentage of anterior vertebral body height			
Preoperative	58.64±6.78	58.13±6.98	0.573ψ
Postoperative 3 months	87.62±2.79	87.57±2.66	
Postoperative 6 months	86.61±1.83	86.39±1.89	
Postoperative 12 months	84.40±1.95 *	84.25±1.97 **	
Percentage of posterior vertebral body height			
Preoperative	86.20±1.71	86.05±1.52	0.662ψ
Postoperative 3 months	92.06±1.60	92.14±1.54	
Postoperative 6 months	91.66±1.35	91.79±1.30	
Postoperative 12 months	89.94±1.60φ	90.05±1.38 φφ	
Spinal canal encroachment (%)			
Preoperative	30.36±5.05	32.25±5.80	0.000ψ
Postoperative 3 months	5.92±1.47	3.45±1.03	
Postoperative 6 months	6.59±1.36	5.04±0.87	
Postoperative 12 months	8.65±1.12#	5.96±0.62##	

^*,*,φ,#^Using the paired *t* test; ^ψ^Using the repeated measures analysis of variance (RMANOVA). The corresponding *p* values of kyphosis Cobb angle, vertebral anterior margin height percentage, posterior margin height percentage, and spinal canal invasion rate in the two groups were compared with the preoperative comparison. ^*^p<0.05, ^**^p<0.05, ^*^p<0.05, ^**^p<0.05, ^φ^*p*<0.05, ^φφ^*p*<0.05, ^#^*p*<0.05, ^##^<0.05

### Complications

There were no wound infections of implant failures in the two groups. In the CT-assisted group, there were 6 cases (10.7%) of adjacent segment degeneration (ASD) and 5 cases (8.9%) of mal-union of fractured vertebrae. In the non-CT-assisted group, there were 5 cases (10%) of ASD and 4 cases (8%) of the fractured vertebra mal-union. There was no difference between the groups in terms of complications when analyzed with the chi-squared test (Fisher’s exact test) (*p*>0.05, Table 6).

**Table 6:** Complications

Variable	Non-CT-assisted group (*N*=50)	CT-assisted group (*N*=56)	*p* value
Infection	2(4%)	2(3.6%)	1.000^*^
Implant failure	0	0	/
Adjacent segment degeneration	5(10%)	6(10.7%)	0.904^*^
Mal-union of fractured vertebrae 4(8%)		5(8.9%)	1.000^*^
Total	11(22%)	13(23.2%)	0.881^*^

^*^Using chi-squared test (Fisher’s exact test)

## Discussion

Decompression through lamina fenestration can reveal the posterior edge of the vertebral body. There is a good view and operating space to reduce the fracture fragments that protrude into the spinal canal, allowing the surgeon to perform surgical operations calmly. Of course, many scholars [11] worry about the compression or interference of the spinal cord’s lumbar enlargement, the conus and cauda equina nerve, and aggravation of the injury [12]. We performed spinal canal decompression and bone grafting without disturbing the dura mater from the front and back, and the operating space was relatively wide, which increased the compensation space during decompression operation. Besides, the operation was carefully performed, and the dural sac was gently stretched not to exceed the vertebrae. The spinal canal’s midline (buffered by cerebrospinal fluid) can effectively avoid spinal cord and nerve damage. After spinal canal decompression, unilateral or bilateral facet joints grafting can be carried out. It can be seen from this study that there is no spinal cord or nerve injury or aggravation of the original spinal cord and nerve injury, and the nerve injury has been restored to different degrees, indicating the safety and effectiveness of this surgical method.

We believe that each patient should be guided by the concept of precision medicine (PM) and rapid recovery. With the assistance of the intraoperative CT, we can accurately monitor fracture reduction and change in the spinal canal compromise; By performing limited decompression of unilateral lamina, the vertebral body’s posterior wall was tamped ventrally. In the non-CT assisted group, bilateral limited lamina decompression was performed to obtain sufficient nerve decompression. After distraction and decompression, open the interlaminar space and slightly pull the dural sac to the midline to find the fracture line; it can be directly seen whether the fracture is completely reduced. Use a percussion instrument to reduce the residual bone block that protruded into the canal. If unilateral limited lamina decompression is performed without CT’s assistance, an “L”-shaped percussion instrument can be used to help reduce the contralateral fracture; however, this may make the decompression effect of the spinal canal unsatisfactory. Lee [13] and Kuner [14] found that the incidence of spinal canal encroachment was an independent risk factor for neurologic symptoms in thoracolumbar burst fractures, and the incidence of spinal canal compromise was directly correlated with the extent of neurologic injury. In other words, a high degree of post-operative spinal canal encroachment may inadequately alleviate neural compression and increase the risk of revision surgery [15, 16], so we tried our best to reduce the posterior to a nearly normal position. Unilateral interlaminar decompression without CT assistance is theoretically feasible, but its clinical effects require more studies to provide evidence-based results.

At present, there is a lack of literature on the treatment of A3 lumbar burst fracture with CT-assisted interlaminar fenestration and decompression. This is a retrospective comparative study of CT-assisted and non-CT-assisted single-segment A3 lumbar burst fractures with limited decompression. In this study, there was no significant difference in the perioperative index, Cobb angle, vertebral body height, and the incidence of post-operative complications between the two groups. This shows that the two methods are equally safe and effective in clinical application. Compared with the non-CT-assisted group, the post-operative spinal canal encroachment and VAS score decreased, while the SF-12 scores, JOA scores, and ASIA grade increased in the CT-assisted group. Some scholars previously had analyzed that multi-slice spiral CT scan with three-dimensional reconstruction can provide a more direct view and real-time three-dimensional images, compensating for the deficiencies of C-arm fluoroscopy and the mobile C-arm CT [17]. Multi-planar reconstruction (MPR) is a three-dimensional layer block selected from acquired data from different angles or along a certain plane. It can be reconstructed with coronal, sagittal, or arbitrarily angled planes to fully display the fracture line and the fracture’s displacement, reducing the cross-sectional false-negative phenomenon [18, 19]. Intraoperative scanning was performed to track and check whether the spinal canal’s posterior wall is reduced close to the normal and determine the residual bone mass position, which plays a decisive guiding role in the operation, thus avoiding limited bilateral decompression. Thorough and effective spinal cord decompression can reduce the post-operative spinal canal encroachment and VAS score, increase post-operative JOA score and HRQoL, and improve long-term neurological function recovery.

In our study, patients were treated with short-segment transpedicular screw fixation [20, 21]. No spondylolisthesis or implant failure [22–26] was observed during follow-up. Composite artificial bone graft was used in either group, and no nerve injury, deformity, neurologic symptoms, and pain deterioration [27–29] were observed. The incidence of fractured vertebrae mal-union was 8.9% in the CT-assisted cohort and 8% in the non-CT-assisted cohort, which was lower than that reported by Bredin et al. [30]. The incidence of adjacent segmental degeneration was 10.7% and 10%, respectively, which was lower than that of (47%) reported by Schaeren et al. [31]. This may be due to our study’s small sample size, short follow-up time, poor vertebral healing, and slow progression of adjacent level degeneration. Nine patients with post-operative mal-union of fractured vertebrae healed well after a second operation with enough artificial bone grafts. Four patients with post-operative wound infection and five patients with post-operative fever were cured after symptomatic treatment. There was no implant failure, which may have been secondary to the small sample size. Therefore, increasing the sample size and long-term follow-up may yield more detailed findings.

This study explores CT’s application in type A3 lumbar burst fracture surgery and tries to reduce patients’ surgical trauma to the possible minimum level. Meanwhile, the study also has some limitations: (1) the sample size is limited, and further research is needed with a bigger sample size; and (2) the follow-up time is relatively short, and long-term follow-up is needed to evaluate the complications such as adjacent segmental degeneration fully. (3) significant progress has been made in image-guided surgery using a navigation system over the last few decades, such as an intraoperative 3D CT-based navigation system (O-arm). The new intraoperative navigation system will be the main focus of our future studies. (4) There is a lack of biomechanical study on pedicle internal fixation (4 nails and 2 rods) across short segments of injured vertebrae. (5) Multiple CT scans are needed during the operation, and the radiation damage to patients and surgical staff is inevitable and cannot be ignored, so we should communicate with patients before the operation. (6) This study is a retrospective clinical study, which needs to be further explored by randomized controlled trials.

## Conclusion

Computed tomography has a robust guiding effect on limited interlaminar decompression and fracture block reduction in the operation of single-segment A3 lumbar burst fracture, which improves the science and reliability of the treatment of A3 lumbar burst fracture. This technique can reduce surgical trauma and achieve satisfactory surgical results and provide patients with a high quality of life and good long-term recovery of neurological function. It is a technique worth popularizing and has a particular prospect of clinical application.

## Data Availability

The datasets used and/or analyzed during the current study are available from the corresponding author on reasonable request.

## Abbreviations

JOA: Japan Orthopaedic Association Score
ASIA: American Spinal Injury Association Spinal Cord Injury Grade
VAS: Visual analog scale
SF-12: Simplified HRQoL scale
CT: Computed tomography
MRI: Magnetic resonance imaging
PLL: Posterior longitudinal ligament
AP: Anteroposterior
PM: Precision medicine
ASD: Adjacent segment degeneration
